# Factors associated with dietary supplement use among physically active adults in Saudi Arabia: an analytical extension of a population-based study

**DOI:** 10.3389/fnut.2026.1955972

**Published:** 2026-09-29

**Authors:** Majed M. Alhumaid, Maryam A. Alobaid, Mohamed A. Said

**Affiliations:** 1 Department of Physical Education, College of Education, King Faisal University, Al-Ahsa, Saudi Arabia; 2 Higher Institute of Sports and Physical Activity of Kef, University of Jendouba, El Kef, Tunisia

**Keywords:** adverse effects, dietary supplements, health behavior, nutritional label reading, physical activity, physically active adults, Saudi Arabia, supplementation practices

## Abstract

**Background:**

Dietary supplement (DS) use is common among physically active individuals, but evidence on factors associated with DS use and related behaviors in Saudi Arabia remains limited. This study represents the second stage of a two-part investigation among physically active adults in the Eastern Province of Saudi Arabia. Part I described the prevalence and general patterns of DS use, whereas Part II examined factors associated with DS use, supplementation frequency, self-reported adverse effects, and nutritional fact-label reading.

**Methods:**

A cross-sectional study was conducted among 755 physically active adults (379 males and 376 females) recruited by quota sampling. Part II was a secondary analysis of the same dataset and the same participants included in Part I. Data were collected from November 2023 to January 2024. Participants were Saudi nationals aged 18–60 years who had engaged in physical activity for at least 12 consecutive weeks, provided informed consent, and completed the questionnaire. A validated online questionnaire assessed sociodemographic characteristics, physical activity, DS use and practices, self-reported adverse effects, purchase sources, perceived metabolic enhancement (categorized according to the self-reported percentage of improvement), and nutritional fact-label reading. DS use referred to consumption of at least one dietary supplement during the previous year. A four-category multinomial outcome represented sex-specific DS-use status, with female non-users as the reference category. Multinomial and binary logistic regression models were used to identify factors associated with DS use, supplementation frequency, adverse effects, and label-reading behavior.

**Results:**

Overall, 505 participants (66.9%) reported using at least one DS during the previous year. Although DS use was similarly prevalent among men and women, associated factors differed by sex. Among women, DS use was associated with fitness-, strength-, and cardio-oriented activities, whereas among men it was associated with training frequency and employment status. Supplementation frequency was associated with training frequency, age, sex, and post-exercise recovery. Self-reported adverse effects were associated with younger age and internet purchasing, while nutritional fact-label reading was associated with female sex, public-sector employment, and greater perceived metabolic enhancement.

**Conclusions:**

DS use is common among physically active individuals in Saudi Arabia and is associated with sex-specific, behavioral, and sociodemographic characteristics. These findings emphasize the multifaceted nature of supplementation practices and support targeted, evidence-based strategies to promote informed and safe DS use.

## 1 Introduction

Physically active people worldwide, both professional athletes and those who exercise for leisure, frequently use dietary supplements (DSs) to help achieve desired goals. Although prevalence varies by population and definition, significant consumption has been consistently reported among individuals who train frequently (1, 2). DSs are commonly used to improve performance, accelerate recovery, increase muscle mass and volume, reduce body fat, or fill perceived nutritional gaps (3, 4); other motivations include the desire to maintain general health and prevent illness.

Alongside abundant descriptive data, research has increasingly investigated factors associated with DS use using multivariable techniques. According to evidence from Europe and North America, DS use is influenced by sex, age, training intensity, competitive status, body composition objectives, and beliefs about effectiveness (1, 2). Cultural and environmental factors may moderate these associations, emphasizing the importance of region-specific analyses. A recent systematic review and network meta-analysis by Deng et al. (5) further confirmed that protein, β-alanine, and creatine are effective for specific performance outcomes. However, female athletes remain substantially underrepresented in the available literature.

In Saudi Arabia, engagement in leisure-time physical activity (PA) and organized exercise has increased considerably following public health initiatives associated with Vision 2030. The DS market has expanded, and performance-enhancing products have become more accessible during this transition (6). Although regional studies have described the prevalence and patterns of DS use, limited evidence exists regarding independent behavioral, training-related, and cognitive factors associated with dietary supplement use.

Part I of this investigation—published in *Frontiers in Public Health* (7)—provided a descriptive overview of DS use among physically active individuals in the Eastern Province of Saudi Arabia, including prevalence estimates and bivariate associations with demographic and training-related characteristics (e.g., sex, BMI, occupation, household income, educational level, training frequency, and exercise motivation). However, being limited to descriptive and bivariate analyses, the study did not determine which factors were independently associated with DS use after adjustment for potential confounding variables. Consequently, the relative contribution of sociodemographic, training-related, and behavioral factors to DS use remains unclear.

The present analytical phase was conceptually informed by the socioecological model (8, 9), which posits that health-related behaviors are shaped by factors operating at multiple levels. These include individual characteristics, interpersonal influences, organizational contexts, community environments, and policy factors (10). This framework has been extensively applied to understand dietary and PA behaviors (11, 12). In this study, the framework was used to organize candidate predictors into conceptually related domains rather than to test a multilevel statistical model.

Consistent with this perspective, Al Turki et al. (6) reported that approximately 58% of 477 Saudi adults had used at least one DS during the previous year, with vitamins the most reported products (81%). Female sex and higher educational attainment independently predicted vitamin use. Common motivations for use included symptom relief (24%), health promotion (19%), and improvement of physical appearance (10%), while nearly three-quarters of participants relied on informal sources of information when making supplementation decisions.

Building on these findings, the present study employed multivariable regression analyses to investigate the independent associations of sociodemographic, training-related, and behavioral characteristics with DS use and selected supplementation behaviors among physically active adults in the Eastern Province of Saudi Arabia.

The objectives of this analytical phase were to:

1. Identify the sociodemographic, training-related, and behavioral factors independently associated with DS use among physically active adults in the Eastern Province of Saudi Arabia and determine whether these associations differ according to sex.
2. Examine factors independently associated with supplementation frequency among DS users.
3. Determine factors independently associated with self-reported side effects among supplement users.
4. Identify the factors that are independently associated with the behavior of reading nutritional fact labels among supplement users.

This analytical phase extends the descriptive findings reported in Part I by identifying independent correlates of DS use and related supplementation behaviors. The findings may contribute to the development of targeted educational initiatives, evidence-informed public health interventions, and strategies promoting safer DS practices.

### 1.1 Research questions

Based on the descriptive findings of Part I (7), the present study addressed the following research questions:

1. Which sociodemographic, training-related, and behavioral factors are independently associated with DS use among physically active adults in the Eastern Province of Saudi Arabia, and do these associations differ according to sex?
2. Which factors are independently associated with supplementation frequency among DS users?
3. Which factors are independently associated with self-reported side effects among supplement users?
4. Which factors are independently associated with nutritional fact label-reading behavior among supplement users?

## 2 Methods

### 2.1 Study design

This study represents the analytical phase of a previously published cross-sectional survey investigating DS use among physically active adults in the Eastern Province of Saudi Arabia. Part I (7) described the study design, participant recruitment, questionnaire development, data collection procedures, and descriptive findings. The present study is a secondary analysis of the same dataset and the same 755 participants, and no additional data were collected or modifications made to the validated questionnaire. The original data collection was conducted over 3 months, from November 2023 to January 2024.

Reporting of this study conforms to the Strengthening the Reporting of Observational Studies in Epidemiology (STROBE) Statement (13).

### 2.2 Sampling strategy

A non-probability quota sampling strategy was employed because no comprehensive sampling frame exists for physically active adults in the Eastern Province of Saudi Arabia. Recreational exercisers and gym members are not registered in a centralized database, making probability-based sampling impractical. Quota sampling was therefore used to ensure adequate representation across predefined sociodemographic strata while maintaining operational feasibility.

The primary quotas were defined by sex (male/female) and age group (18–29, 30–44, and ≥45 years). Stratum-specific targets were established *a priori* based on aggregated membership statistics obtained from partnering fitness clubs and gyms across the four recruitment cities. The resulting target proportions were 24% for males aged 18–29 years, 23% for males aged 30–44 years, 4% for males aged ≥45 years, 31% for females aged 18–29 years, 15% for females aged 30–44 years, and 4% for females aged ≥45 years. These ratios were applied to the overall target sample of 330 participants, calculated using Cochran's method (Part I).

City-specific quotas were additionally allocated across the four primary recruitment municipalities—Dammam, Khobar, Qatif, and Al-Ahsa—based on their proportional population sizes according to the latest official statistics from the General Authority for Statistics. The corresponding population proportions were 39.8%, 17.1%, 14.4%, and 28.7%, respectively, and were applied to the target sample of 330 participants to determine the geographic targets.

Participants were recruited through fitness clubs and gyms, social media platforms including X, Instagram, and WhatsApp, supplement stores, and community events in Dammam, Khobar, Qatif, and Al-Ahsa. The target and achieved numbers for the sex-by-age quotas are presented in Supplementary Table S1, and the geographic target and achieved distributions are presented in Supplementary Table S2. This approach is commonly adopted in health behavior research when sampling frames are unavailable and subgroup representation is important (14). Although quota sampling improves representation of selected subgroups, it does not permit full population-level inference; therefore, the findings should be interpreted cautiously when generalized beyond the study population (15).

#### 2.2.1 Eligibility and participant flow

Eligibility criteria were those specified in Part I: participants were required to be Saudi nationals, aged 18–60 years old, have engaged in PA for at least 12 consecutive weeks, provide informed consent, and complete the questionnaire. Individuals with chronic conditions that hindered PA were excluded.

In all, 793 questionnaires were returned. Initial screening rejected 38 questionnaires due to incomplete responses (< 90%), non-serious or patterned responses, or failure to meet PA eligibility criteria. The final analytical sample therefore comprised 755 participants. No additional exclusions were made for the present secondary analyses.

### 2.3 Questionnaire structure

The questionnaire has been fully described in Part I (7), including its development, translation and cultural adaptation, content validity, and test–retest reliability. Briefly, it consisted of two major sections.

#### 2.3.1 Part I. Sociodemographic and socioeconomic characteristics

This section comprised eight core demographic and socioeconomic items assessing participants' demographic and socioeconomic characteristics, including sex, age, self-reported weight and height, marital status, educational attainment, employment status, and household income. Body mass index (BMI) was calculated as weight (kg) divided by height squared (m^2^).

#### 2.3.2 Part II. Dietary supplement use and behavioral characteristics

This section comprised four domains containing 14 structured items. The first domain assessed PA characteristics, including exercise frequency, primary activity type, and exercise motivation. The second domain evaluated DS use, including user status, supplementation objectives, frequency of use, perceived benefits, and self-reported physiological changes following supplementation. The third domain assessed types of DSs used and self-reported adverse effects. The fourth domain examined supplement selection practices, which included the reasons for product choice, the sources of purchase, the behavior of reading nutritional fact labels, and the willingness to recommend supplements to others.

Most questions were multiple-choice items to facilitate standardized data collection, while a limited number included an “Other” response option.

### 2.4 Measurement of study variables

Physical activity was operationalized according to the eligibility criterion used in Part I: participants were considered physically active if they reported consistent participation in PA for at least 12 consecutive weeks. The sample population included members of fitness clubs and gyms, as well as independent exercisers. This operational definition was used to define the study population, not to construct a continuous PA score.

DS use was defined as self-reported use of at least one dietary supplement during the previous 12 months. Supplementation frequency was assessed using a five-category ordinal variable. Exercise motivations, supplementation objectives, purchase sources, and beliefs regarding dietary supplements were measured as categorical variables. Self-perceived metabolic enhancement following supplementation was assessed using a four-category ordinal scale: (1) no perceived positive change, (2) >10% improvement, (3) >20% improvement, and (4) >30% improvement. For regression analyses, these categories were entered as categorical predictors, with no perceived positive change serving as the reference category.

#### 2.4.1 Outcome variables

The primary outcome was self-reported DS use, coded as a binary variable (0 = non-user; 1 = user). For the primary multinomial analysis, DS-use status was combined with sex to form a four-category outcome: male non-user, male user, female non-user, and female user, with female non-users serving as the reference category. Consequently, distinct regression models for each sex were not constructed, and sex was not used as independent variable in this primary model. The interaction terms involving sex and other explanatory variables were not examined as sex was directly included in the multinomial outcome.

Among DS users, three secondary outcomes were examined:

- Supplementation frequency;
- Self-reported side effects (yes/no); and
- Nutritional fact label-reading behavior (yes/no).

#### 2.4.2 Independent variables

The training-related domain included training frequency, primary activity type, and primary motivation for exercise. The behavioral domain included dietary supplement-related characteristics, including supplementation frequency, supplementation objectives, beliefs regarding dietary supplement effectiveness, purchase source, nutritional facts label-reading behavior, and self-reported side effects, as applicable to the specific regression model.

For each regression model, candidate variables were defined *a priori* according to their conceptual relevance to the specific outcome and were informed by the variables collected in the questionnaire and the bivariate findings reported in Part I. The *p* < 0.20 criterion was used as a liberal preliminary screening criterion rather than as the sole basis for variable inclusion. Final model specification also considered conceptual relevance, adequate cell frequencies, and model stability.

### 2.5 Statistical analysis

All analyses were performed using IBM SPSS Statistics version 26 (IBM Corp., Armonk, NY, USA). Descriptive statistics, data screening procedures, and bivariate analyses were reported previously in Part I (7). The present study focused exclusively on multivariable regression analyses to identify factors independently associated with DS use and related supplementation behaviors.

No missing data were present among participants included in the final analytical dataset. Of the 793 questionnaires returned, 38 were excluded during the preliminary data assessment, resulting in 755 eligible participants (250 DS non-users and 505 users). All 755 participants had complete data for the variables included in the primary multinomial logistic regression model, and all 505 DS users had complete data for the variables included in the subsequent regression models.

A multinomial logistic regression model was first constructed using a four-category outcome representing sex-specific supplement-use status (male non-users, male users, female non-users, and female users), with female non-users serving as the reference category. The model included eight prespecified explanatory variables: age, BMI, occupation, household income, educational attainment, training frequency, primary exercise motivation, and primary activity type. These variables yielded 19 predictor parameters per non-reference outcome contrast. Because the multinomial outcome had three non-reference contrasts, the likelihood-ratio test for the fitted model had 57 degrees of freedom (19 × 3).

Among dietary supplement users (*n* = 505), a multinomial logistic regression model was developed to examine supplementation frequency, which comprised five outcome categories: daily supplementation, weekly supplementation, supplementation according to training intensity, supplementation within the recommended dose, and meal replacement, with daily supplementation as the reference category. The model included age, sex, education, frequency of training, and dietary supplement-related predictors described above, together with four supplementation objectives and three supplementation beliefs. These predictors yielded 13 estimated predictor parameters per non-reference outcome contrast. With four non-reference contrasts, the likelihood-ratio test for the fitted model had 52 degrees of freedom (13 × 4).

Binary logistic regression models were used to examine self-reported side effects and nutritional facts label-reading behavior. The side-effects model included 505 supplement users and 19 estimated predictor parameters: one each for age, sex, and training frequency; three each for educational attainment and purchase source; four each for supplementation frequency; and six for the individual dietary supplement-type indicators. The likelihood-ratio test for the fitted model therefore had 19 degrees of freedom.

The label-reading model also included 505 supplement users. The model incorporated age, sex, educational attainment, training frequency, supplementation frequency, self-perceived metabolic enhancement following supplementation, purchase source, and self-reported side effects. These predictors yielded 17 estimated predictor parameters, corresponding to 17 degrees of freedom for the model likelihood-ratio test.

Before model construction, several categorical variables were collapsed to avoid sparse cell frequencies. Recoding was performed before regression modeling and was based on observed category frequencies. Sparse categories were merged with an adjacent preceding or following category according to the observed sample size, while retaining the substantive meaning and, where applicable, the order of the original response categories.

Household income was reduced from six original categories to five, with the 15,000–19,999 SAR and ≥20,000 SAR categories combined into a single ≥15,000 SAR category. Educational attainment was reduced from five categories to four, with intermediate and secondary education combined into secondary education or less. Training frequency was converted to an estimated number of training sessions per day for multivariable regression analyses. The corresponding numerical values were 0.03 sessions/day for once per month, 0.14 sessions/day for once per week, 0.13 sessions/day for ≥4 times per month, 0.71 sessions/day for ≥4 times per week, and 1.00 sessions/day for daily/competitive athletes. Exercise motivation was recategorized by combining medical reasons and social interaction into a single category. Similarly, the original PA variable—which included more than nine activity types, in addition to “Other” and “More than one activity”—was consolidated into three categories: Fitness/Strength/Cardio, Aerobic exercise, and Stationary cycling. The objective of DS use was also recategorized by combining “Weight loss” and “Reduce body fat” into a single “Weight management” category.

These recategorizations were specified before regression modeling to address sparse cell frequencies and improve the stability and interpretability of parameter estimates.

Candidate variables were first evaluated based on their theoretical significance and the associated bivariate results. Variables exhibiting *p* < 0.20 in bivariate analyses were deemed suitable for multivariable modeling; however, this threshold was used as a screening criterion and was not applied as an exclusive automatic rule. Conceptually important variables were retained where justified, and final model specification considered parsimony, adequate cell frequencies, and model stability.

Model adequacy for multinomial logistic regression was evaluated using likelihood-ratio tests and Pearson goodness-of-fit statistics. Binary logistic regression models were assessed using the Hosmer–Lemeshow goodness-of-fit test, with *p* > 0.05 indicating acceptable model fit. Model performance was summarized using Nagelkerke's pseudo-*R*^2^. Multicollinearity was evaluated using variance inflation factors (all < 5), and the assumption of linearity in the logit for continuous predictors was verified before model estimation. Adjusted odds ratios (ORs) with 95% confidence intervals (CIs) are reported, and statistical significance was established at a two-sided α level of 0.05.

### 2.6 Ethical considerations

The study was conducted in accordance with the ethical principles of the Declaration of Helsinki (16). Ethical approval for the overall project was obtained from the Research Ethics Committee at King Faisal University, Saudi Arabia (KFU-REC-2023-OCT-ETHICS1589; approved 13 October 2023). Details of the ethical approval and informed consent procedures were reported in Part I (7). The present manuscript constitutes Part II of the project and uses the anonymized dataset collected under this ethical approval. No additional ethical approval was required for Part II.

## 3 Results

### 3.1 Participant characteristics

A total of 755 physically active adults participated in the study, of whom 505 (66.9%) reported consuming at least one DS during the previous year. Participant characteristics according to sex-specific DS-use status are presented in Table 1.

**Table 1:** Participant characteristics according to sex-specific dietary supplement-use status (*N* = 755).

Variable	Category	Male non-users (*n* = 132)	Male users (*n* = 250)	Female non-users (*n* = 118)	Female users (*n* = 255)	Total (*N* = 755)
Age (years)	Mean ± SD	31.23 ± 8.34	30.88 ± 7.66	27.80 ± 7.98	28.41 ± 8.03	**29.63** **±8.06**
BMI (kg/m^2^)	Mean ± SD	25.57 ± 5.58	26.59 ± 6.17	24.45 ± 4.69	24.97 ± 5.25	**25.53** **±5.60**
Occupation	Unemployed	11 (8.3)	23 (9.2)	30 (25.4)	65 (25.5)	**129 (17.1)**
	Government	58 (43.9)	96 (38.4)	12 (10.2)	30 (11.8)	**196 (26.0)**
	Private sector	45 (34.1)	94 (37.6)	38 (32.2)	71 (27.8)	**248 (32.8)**
	Student	18 (13.6)	37 (14.8)	38 (32.2)	89 (34.9)	**182 (24.1)**
Monthly household income (SAR)	No income	18 (13.6)	36 (14.4)	26 (22.0)	64 (25.1)	**144 (19.1)**
	< 5,000	19 (14.4)	39 (15.6)	21 (17.8)	41 (16.1)	**120 (15.9)**
	5,000–9,999	26 (19.7)	59 (23.6)	33 (28.0)	59 (23.1)	**177 (23.4)**
	10,000–14,999	32 (24.2)	54 (21.6)	15 (12.7)	42 (16.5)	**143 (18.9)**
	15,000–19,999	22 (16.7)	31 (12.4)	12 (10.2)	25 (9.8)	**90 (11.9)**
	≥20,000	15 (11.4)	31 (12.4)	11 (9.3)	24 (9.4)	**81 (10.7)**
Education level	Intermediate	0 (0.0)	2 (0.8)	5 (4.2)	4 (1.6)	**11 (1.5)**
	Secondary	26 (19.7)	50 (20.0)	24 (20.3)	59 (23.1)	**159 (21.1)**
	Diploma	24 (18.2)	60 (24.0)	10 (8.5)	26 (10.2)	**120 (15.9)**
	Bachelor's	66 (50.0)	118 (47.2)	69 (58.5)	142 (55.7)	**395 (52.3)**
	Postgraduate	16 (12.1)	20 (8.0)	10 (8.5)	24 (9.4)	**70 (9.3)**
Training frequency	Once per month	1 (0.8)	5 (2.0)	17 (14.4)	36 (14.1)	**59 (7.8)**
	Once per week	3 (2.3)	13 (5.2)	6 (5.1)	11 (4.3)	**33 (4.4)**
	≥4 times per month	11 (8.3)	20 (8.0)	19 (16.1)	45 (17.6)	**95 (12.6)**
	≥4 times per week	92 (69.7)	154 (61.6)	52 (44.1)	108 (42.4)	**406 (53.8)**
	Competitive athlete	25 (18.9)	58 (23.2)	24 (20.3)	55 (21.6)	**162 (21.5)**
Primary motivation for exercise	Enjoyment	16 (12.1)	51 (20.4)	28 (23.7)	39 (15.3)	**134 (17.7)**
	Aesthetic appearance	22 (16.7)	45 (18.0)	15 (12.7)	38 (14.9)	**120 (15.9)**
	Medical reasons	18 (13.6)	18 (7.2)	10 (8.5)	36 (14.1)	**82 (10.9)**
	Financial gain	1 (0.8)	6 (2.4)	2 (1.7)	4 (1.6)	**13 (1.7)**
	Social interaction	2 (1.5)	1 (0.4)	0 (0.0)	4 (1.6)	**7 (0.9)**
	Multiple motivations	73 (55.3)	129 (51.6)	63 (53.4)	134 (52.5)	**399 (52.8)**
Primary activity type	Fitness/Strength/Cardio	48 (36.4)	36 (14.4)	24 (20.3)	112 (43.9)	**220 (29.1)**
	Aerobic exercise	57 (43.2)	147 (58.8)	67 (56.8)	112 (43.9)	**383 (50.7)**
	Stationary cycling	27 (20.5)	67 (26.8)	27 (22.9)	31 (12.2)	**152 (20.1)**

Values are presented as mean ± standard deviation (SD) for continuous variables and as number (percentage) for categorical variables. BMI, body mass index; SAR, Saudi Arabian Riyal. Detailed descriptive statistics and bivariate comparisons were reported previously in Part I (7).

As described in Part I (7), the study population comprised a relatively young cohort of physically active adults. In the present study, participant characteristics were reorganized according to the four outcome categories used in the multinomial logistic regression analysis (male non-users, male users, female non-users, and female users) to facilitate comparison across the analytical groups.

The mean age of male DS users was 30.88 ± 7.66 years compared with 28.41 ± 8.03 years among female DS users; corresponding mean BMI values were 26.59 ± 6.17 kg/m^2^ and 24.97 ± 5.25 kg/m^2^, respectively. Across all four groups, most participants held a bachelor's degree and reported engaging in PA more than four times per week. Government and private-sector employment predominated among men, whereas women were more frequently students or unemployed.

### 3.2 Supplementation practices among dietary supplement users

Dietary supplement practices among the 505 DS users are summarized in Table 2. Daily supplementation was the most frequently reported pattern of use (39.0%), followed by adherence to the recommended daily dose (22.4%), meal replacement (14.7%), weekly supplementation (12.7%), and supplementation according to training intensity (11.3%).

**Table 2:** Dietary supplement practices, perceptions, and self-reported adverse effects among dietary supplement users (*N* = 505).

Variable	Category	Male *n* (%)	Female *n* (%)	Total *n* (%)
Pattern of dietary supplement use	Daily	**116 (46.4)**	**81 (31.8)**	197 (39.0)
	Do not exceed the recommended dose	**45 (18.0)**	**68 (26.7)**	113 (22.4)
	Weekly	**30 (12.0)**	**34 (13.3)**	64 (12.7)
	According to training intensity	**28 (11.2)**	**29 (11.4)**	57 (11.3)
	Used as meal replacement	**31 (12.4)**	**43 (16.9)**	74 (14.7)
Self-perceived metabolic enhancement following supplementation	No perceived change	**22 (8.8)**	**48 (18.8)**	70 (13.9)
	Slight improvement (>10%)	**83 (33.2)**	**115 (45.1)**	198 (39.2)
	Moderate improvement (>20%)	**81 (32.4)**	**57 (22.4)**	138 (27.3)
	Marked improvement (>30%)	**64 (25.6)**	**35 (13.7)**	99 (19.6)
Reads the product label before using dietary supplements	Yes	**204 (81.6)**	**218 (85.5)**	422 (83.6)
	No	**46 (18.4)**	**37 (14.5)**	83 (16.4)
Would recommend dietary supplements to others	Yes	**187 (74.8)**	**205 (80.4)**	392 (77.6)
	No	**24 (9.6)**	**28 (11.0)**	52 (10.3)
	Uncertain	**39 (15.6)**	**22 (8.6)**	61 (12.1)
Primary purchase source	Authorized distributor	**124 (49.6)**	**64 (25.1)**	188 (37.2)
	Pharmacy	**38 (15.2)**	**124 (48.6)**	162 (32.1)
	Internet	**72 (28.8)**	**54 (21.2)**	126 (25.0)
	Friends	**18 (7.2)**	**11 (4.3)**	29 (5.7)
Objectives of dietary supplement use	Compensate for nutritional deficiencies	**124 (49.6)**	**163 (63.9)**	287 (56.8)
	Promote muscle building	**167 (66.8)**	**97 (38.0)**	264 (52.3)
	Increase muscular strength	**125 (50.0)**	**73 (28.6)**	198 (39.2)
	Enhance post-exercise recovery	**90 (36.0)**	**69 (27.1)**	159 (31.5)
	Weight loss	**45 (18.0)**	**78 (30.6)**	113 (22.4)
	Reduce body fat	**42 (16.8)**	**37 (14.5)**	79 (15.6)
Beliefs regarding dietary supplements	Provide nutrients not adequately obtained from diet	**89 (35.6)**	**90 (35.3)**	179 (35.4)
	Increase energy levels	**88 (35.2)**	**84 (32.9)**	172 (34.1)
	Promote muscle growth	**87 (34.8)**	**75 (29.4)**	162 (32.1)
	Assist weight loss when combined with diet	**40 (16.0)**	**39 (15.3)**	79 (15.6)
Reasons for choosing dietary supplements	Information from online sources or friends	**135 (54.0)**	**122 (47.8)**	257 (50.9)
	Recommendation from a healthcare or nutrition professional	**112 (44.8)**	**140 (54.9)**	252 (49.9)
	Affordable price	**88 (35.2)**	**63 (24.7)**	151 (29.9)
	Brand sponsorship	**20 (8.0)**	**22 (8.6)**	42 (8.3)
Types of dietary supplements used	Vitamins	**127 (50.8)**	**165 (64.7)**	292 (57.8)
	Protein supplements	**133 (53.2)**	**98 (38.4)**	231 (45.7)
	Antioxidants	**75 (30.0)**	**59 (23.1)**	134 (26.5)
	Muscle-building protein supplements	**83 (33.2)**	**39 (15.3)**	122 (24.2)
	Energy supplements	**77 (30.8)**	**41 (16.1)**	118 (23.4)
	Amino acid supplements	**85 (34.0)**	**31 (12.2)**	116 (23.0)
	Fat burners	**31 (12.4)**	**37 (14.5)**	68 (13.5)
Self-reported adverse effects	No adverse effects	**168 (67.2)**	**158 (62.0)**	326 (64.6)
	Nausea	**25 (10.0)**	**48 (18.8)**	73 (14.5)
	Diarrhea	**15 (6.0)**	**15 (5.9)**	30 (5.9)
	Change in body odor	**16 (6.4)**	**14 (5.5)**	30 (5.9)
	Symptoms suggestive of toxicity	**8 (3.2)**	**9 (3.5)**	17 (3.4)
	Chest pain	**4 (1.6)**	**8 (3.1)**	12 (2.4)
	Vomiting	**5 (2.0)**	**8 (3.1)**	11 (2.2)
	Muscle cramps	**4 (1.6)**	**4 (1.6)**	8 (1.6)
	Constipation	**1 (0.4)**	**4 (1.6)**	5 (1.0)
	Allergic reaction	**3 (1.2)**	**1 (0.4)**	4 (0.8)

Values are presented as number (percentage). Multiple responses were permitted for supplementation objectives, beliefs, reasons for choosing dietary supplements, supplement types, and self-reported adverse effects; therefore, percentages for these variables may exceed 100%. Percentages were calculated using the total number of dietary supplement users (*N* = 505) as the denominator.

Overall, 86.1% of users reported some degree of perceived metabolic enhancement following DS use. Most participants indicated that they routinely read nutritional fact labels before using DSs (83.6%), and more than three-quarters (77.6%) reported that they would recommend DSs to others.

Authorized distributors (37.2%) and pharmacies (32.1%) were the principal sources of DSs, although one-quarter of participants purchased supplements through internet retailers (25.0%). The most frequently reported supplementation objectives were compensating for nutritional deficiencies (56.8%) and promoting muscle building (52.3%). Vitamins (57.8%) and protein supplements (45.7%) were the most commonly consumed products.

Overall, approximately one-third of supplement users reported at least one self-reported adverse effect (35.4%), with nausea being the most frequently reported symptom (14.5%).

### 3.3 Factors independently associated with dietary supplement use

Multinomial logistic regression was performed to identify factors independently associated with dietary supplement-use status, using female non-users as the reference category. The model included 755 participants and 57 estimated predictor parameters across the three non-reference outcome contrasts. The model demonstrated acceptable goodness of fit and explained 31.4% of the variance (Nagelkerke *R*^2^ = 0.314; Table 3).

**Table 3:** Multinomial logistic regression: factors independently associated with dietary supplement use.

Comparison group	Predictor	OR	95% CI	*p*-value
Male non-users	Training frequency	6.84	2.77–16.86	< 0.001
	Job: public sector (vs. student)	14.00	4.48–43.74	< 0.001
	Job: private sector (vs. student)	2.81	1.15–6.88	0.024
Male users	Training frequency	4.50	2.12–9.57	< 0.001
	Job: public sector (vs. student)	10.92	3.97–30.08	< 0.001
	Job: private sector (vs. student)	2.71	1.27–5.79	0.010
Female users	Activity: fitness/strength/cardio (vs. stationary cycling)	3.79	1.89–7.58	< 0.001

B, regression coefficient (log-odds coefficient); OR, odds ratio; CI, confidence interval. Female non-users served as the reference category for the dependent variable. Reference categories for categorical predictors are shown explicitly in the Predictor column. Adjusted ORs with 95% CIs are presented. Only predictors retained in the final model are displayed; candidate variables not retained in the final model are omitted for clarity.

Overall likelihood ratio tests identified training frequency, occupation, and primary activity type as the only variables independently associated with DS-use status (all *p* < 0.001). Age, household income, educational attainment, and primary exercise motivation were not independently associated with DS use.

Compared with female non-users, female DS users had significantly higher odds of primarily engaging in fitness-, strength-, or cardio-oriented activities rather than stationary cycling (OR = 3.79, 95% CI: 1.89–7.58, *p* < 0.001).

Compared with female non-users, male non-users had significantly higher odds of reporting higher training frequency (OR = 6.84, 95% CI: 2.77–16.86, *p* < 0.001), public-sector employment (OR = 14.00, 95% CI: 4.48–43.74, *p* < 0.001), and private-sector employment (OR = 2.81, 95% CI: 1.15–6.88, *p* = 0.024). Similarly, compared with female non-users, male DS users had significantly higher odds of reporting higher training frequency (OR = 4.50, 95% CI: 2.12–9.57, *p* < 0.001), public-sector employment (OR = 10.92, 95% CI: 3.97–30.08, *p* < 0.001), and private-sector employment (OR = 2.71, 95% CI: 1.27–5.79, *p* = 0.010).

### 3.4 Factors associated with supplementation frequency

Among the 505 participants who reported dietary supplement use, multinomial logistic regression was performed to identify factors associated with supplementation frequency. The outcome variable comprised five categories: daily (39.0%, reference category), weekly (12.7%), according to training intensity (11.3%), within the recommended dose (22.4%), and to replace main meals (14.7%). The model included 13 predictor terms, corresponding to 52 estimated parameters across the four non-reference outcome contrasts. The model demonstrated good fit and explained 20.9% of the variance (Nagelkerke *R*^2^ = 0.209; Table 4).

**Table 4:** Multinomial logistic regression analysis of factors associated with supplementation frequency among dietary supplement users.

Comparison	Predictor	OR	95% CI	*p*-value
Weekly (vs. daily)	Training frequency (per unit increase)	0.41	0.18–0.91	0.029
	Sex (male vs. female)	0.46	0.27–0.79	0.005
	Objective: compensate for nutritional deficiencies (No vs. Yes)	0.55	0.32–0.93	0.027
	Belief: increases energy levels (No vs. Yes)	2.01	1.16–3.49	0.013
	Belief: promotes muscle growth (No vs. Yes)	0.58	0.34–0.99	0.046
According to training intensity (vs. daily)	Training frequency (per unit increase)	0.35	0.14–0.90	0.030
	Objective: promote muscle building (No vs. Yes)	2.38	1.20–4.71	0.013
Within the recommended dose (vs. daily)	Age (per year increase)	0.95	0.91–1.00	0.041
	Belief: provides nutrients not adequately obtained from diet (No vs. Yes)	2.16	1.03–4.52	0.042
Replace main meals (vs. daily)	Sex (male vs. female)	0.49	0.27–0.89	0.019
	Objective: enhance post-exercise recovery (No vs. Yes)	3.00	1.41–6.38	0.004
	Belief: increases energy levels (No vs. Yes)	2.05	1.10–3.82	0.024

OR, odds ratio; CI, confidence interval. Daily supplementation served as the reference category for the dependent variable. Reference categories for categorical predictors are indicated in the table. Adjusted ORs with 95% CIs are presented. Only variables retained in the final multinomial logistic regression model are reported.

Overall likelihood-ratio tests indicated that age, sex, training frequency, and post-exercise recovery were significantly associated with supplementation frequency (all *p* < 0.05). The remaining predictor terms did not show statistically significant overall associations with supplementation frequency.

#### 3.4.1 Demographic characteristics

Age was associated with supplementation frequency. Compared with daily supplementation, each one-year increase in age was associated with a 5% reduction in the odds of supplementation within the recommended dose (OR = 0.95, 95% CI: 0.91–1.00, *p* = 0.041).

Sex differences were also observed. Compared with female users, male users had lower odds of weekly supplementation (OR = 0.46, 95% CI: 0.27–0.79, *p* = 0.005) and meal-replacement supplementation (OR = 0.49, 95% CI: 0.27–0.89, *p* = 0.019), relative to daily supplementation.

#### 3.4.2 Training characteristics

Training frequency was independently associated with supplementation frequency. Compared with daily supplementation, each one-unit increase in training frequency was associated with lower odds of weekly supplementation (OR = 0.41, 95% CI: 0.18–0.91, *p* = 0.029) and supplementing according to training intensity (OR = 0.35, 95% CI: 0.14–0.90, *p* = 0.030).

#### 3.4.3 Supplementation objectives

Participants who supplemented weekly had lower odds of reporting compensation for nutritional deficiencies as a supplementation objective than those who supplemented daily (OR = 0.55, 95% CI: 0.32–0.93, *p* = 0.027). Participants who supplemented according to training intensity had higher odds of not identifying muscle building as a supplementation objective (OR = 2.38, 95% CI: 1.20–4.71, *p* = 0.013). Similarly, participants who used supplements to replace main meals had higher odds of not identifying post-exercise recovery as a supplementation objective (OR = 3.00, 95% CI: 1.41–6.38, *p* = 0.004).

#### 3.4.4 Supplementation beliefs

Participants who supplemented weekly had higher odds of reporting that supplements increase energy levels (OR = 2.01, 95% CI: 1.16–3.49, *p* = 0.013) and lower odds of reporting that supplements promote muscle growth (OR = 0.58, 95% CI: 0.34–0.99, *p* = 0.046), compared with those who supplemented daily.

Participants who supplemented within the recommended dose had higher odds of reporting that supplements provide nutrients not adequately obtained from the diet (OR = 2.16, 95% CI: 1.03–4.52, *p* = 0.042). Finally, participants who used supplements to replace main meals had higher odds of reporting that supplements increase energy levels (OR = 2.05, 95% CI: 1.10–3.82, *p* = 0.024).

### 3.5 Factors associated with self-reported side effects

Among dietary supplement users, 177 participants (35.4%) reported experiencing at least one self-reported side effect. Binary logistic regression was performed to identify factors independently associated with these adverse effects. The analysis included all 505 supplement users and 19 estimated predictor parameters. The model demonstrated acceptable fit and explained 9.4% of the variance (Nagelkerke *R*^2^ = 0.094; Table 5). Three variables remained independently associated with self-reported side effects.

**Table 5:** Binary logistic regression analysis of factors associated with self-reported side effects among dietary supplement users.

Predictor	OR	95% CI	*p*-value
Age (per year)	0.96	0.93–0.98	0.002
Income: middle (vs. highest)	2.23	1.11–4.51	0.025
Source: internet (vs. Authorized distributors)	3.41	1.23–9.45	0.018

OR, odds ratio; CI, confidence interval. The dependent variable was self-reported side effects (Yes = 1, No = 0). Reference categories are indicated for categorical predictors. The purchase-source variable was collapsed into Internet vs. other sources before modeling due to sparse cells. Adjusted ORs with 95% CIs are presented.

#### 3.5.1 Demographic characteristics

Increasing age was associated with a lower likelihood of reporting side effects (OR = 0.96, 95% CI: 0.93–0.98, *p* = 0.002). In contrast, participants with middle household income had significantly greater odds of reporting side effects than those in the highest income category (OR = 2.23, 95% CI: 1.11–4.51, *p* = 0.025).

#### 3.5.2 Purchasing source

Purchasing dietary supplements through internet retailers was independently associated with an increased likelihood of self-reported side effects (OR = 3.41, 95% CI: 1.23–9.45, *p* = 0.018), compared with purchasing from authorized distributors.

No significant associations were observed for sex, educational attainment, supplementation frequency, nutritional fact label-reading behavior, or the remaining purchasing sources (all *p* > 0.05).

### 3.6 Factors associated with nutritional fact label reading

Among DS users, 422 participants (83.6%) reported reading nutritional facts labels before using dietary supplements. Binary logistic regression was performed to identify factors independently associated with label-reading behavior. The analysis included all 505 supplement users and 17 estimated predictor parameters. The model demonstrated acceptable goodness of fit and explained 18.0% of the variance (Nagelkerke *R*^2^ = 0.180; Table 6).

**Table 6:** Binary logistic regression analysis of factors associated with nutritional fact label reading among dietary supplement users.

Predictor	Comparison	OR	95% CI	*p*-value
Sex	Female vs. male	3.25	1.74–6.08	< 0.001
Job	Public sector vs. no job	2.78	1.05–7.36	0.039
Positive effects (>10%)	>10% acceleration vs. no positive change	3.08	1.57–6.03	0.001
Positive effects (>20%)	>20% acceleration vs. no positive change	6.68	2.95–15.12	< 0.001
Positive effects (>30%)	>30% acceleration vs. no positive change	7.43	2.93–18.87	< 0.001

OR, odds ratio; CI, confidence interval. The dependent variable was nutritional facts label reading (Yes = 1, No = 0). Reference categories were male, no job, and no perceived positive metabolic change. Adjusted ORs with 95% CIs are presented.

#### 3.6.1 Demographic characteristics

Women were significantly more likely than men to report reading nutritional fact labels before using DSs (OR = 3.25, 95% CI: 1.74–6.08, *p* < 0.001). Public-sector employment was also independently associated with label-reading behavior compared with unemployment (OR = 2.78, 95% CI: 1.05–7.36, *p* = 0.039).

Age demonstrated a borderline association with label reading (*p* = 0.059), whereas household income, educational attainment, supplementation frequency, and purchasing source were not independently associated with the outcome.

#### 3.6.2 Perceived metabolic enhancement

The perception of metabolic enhancement after using DSs was strongly associated with reading nutritional fact labels. This variable was assessed using a four-category ordinal response: no perceived positive change, >10%, >20%, and >30% perceived metabolic improvement. Compared with participants reporting no perceived metabolic enhancement, progressively greater perceived improvement was associated with progressively higher odds of reading nutritional facts labels, including perceived improvements of >10% (OR = 3.08, 95% CI: 1.57–6.03, *p* = 0.001), >20% (OR = 6.68, 95% CI: 2.95–15.12, *p* < 0.001), and >30% (OR = 7.43, 95% CI: 2.93–18.87, *p* < 0.001).

## 4 Discussion

The present analytical phase extends the descriptive findings—reported in Part I of the study—by identifying the various factors associated with dietary supplement use among physically active adults in Saudi Arabia, particularly highlighting gender-specific use patterns, supplementation frequency, self-reported side effects, and the behavior of reading nutritional labels. To our knowledge, this is one of the few studies conducted among physically active adults in Saudi Arabia to simultaneously examine multiple dimensions of DS use using multivariable analyses. Nevertheless, because the study was cross-sectional, the observed associations should not be interpreted as causal relationships.

### 4.1 Factors associated with dietary supplement use

Despite a similar prevalence of supplementation among men (66.0%) and women (67.8%), we identified separate factors that influence DS use among physically active individuals. In comparison to the 58% prevalence reported in the general Saudi adult population, 66.9% of participants reported using DSs (6). Consistent with earlier research linking regular exercise to increased supplement usage, this finding indicates that supplement use is more prevalent among physically active individuals.

Our data indicated that while DS use was approximately equal between men and women, the factors that influenced this behavior were very different. This contradicts the results of Al Turki et al. (6), who reported that among Saudis as a whole, female sex was the strongest predictor of vitamin consumption. These disparities may arise from dissimilarities in the research populations. The current study specifically recruited physically active individuals, while Al Turki et al. (6). sampled adults regardless of their PA levels. Our group may have included a lower level of sex disparity than the general population due to shared exercise-related behaviors and access to information on sports nutrition. However, our cross-sectional design precludes any conclusions about cause and effect, and so caution is necessary when interpreting these results.

Following adjustment for potential confounding variables, DS use among women was not independently associated with training frequency. Conversely, activity type remained independently associated with DS use, indicating that the type of PA may be more strongly associated with supplement use than training frequency in this population. Nonetheless, because of the cross-sectional design, it cannot be determined whether specific types of activity influence supplement use or whether women who use supplements are more likely to engage in these activities. Deng et al. (5) reported that protein supplementation was associated with improvements in muscular strength, while creatine and β-alanine were associated with improvements in sprint and jump performance, among other performance outcomes. However, none of the supplements examined consistently increased muscle mass (5). These findings provide a possible context for the observed association between fitness-, strength-, and cardio-oriented activities and DS use, although the specific reasons underlying this association were not assessed in the present study.

A recent comprehensive analysis of dietary plans for women who participate in sports and other PA (17) supports our findings. The study found that certain DSs—including protein, creatine, caffeine, and β-alanine—could potentially enhance performance or aid in recovery in certain scenarios. Nevertheless, the authors noted that women are still underrepresented in sports nutrition research, limiting the reliability of the findings and emphasizing the necessity for advice tailored by sex.

After accounting for possible confounders, DS use among women was not independently correlated with training frequency. This result suggests that supplementing behavior may be better predicted by participation in structured fitness and resistance-training activities than by exercise volume alone. However, the cross-sectional design of the present study does not allow us to assess if these activities promote supplement use or if these exercise modalities are preferred by supplement users.

In contrast, men's DS consumption was not independently correlated with activity type. Compared to female non-users, men who used supplements and those who did not had higher training frequencies and were more likely to work in the public or private sectors. The fact that these trends were represented in both DS-using and non-using males suggests that they are more indicative of general demographic and exercise-related differences than of reasons that motivate supplementation in particular. Other potentially relevant factors, such as nutrition knowledge, access to professional nutritional guidance, social influences, and exposure to supplement marketing, were not measured in the present study and therefore remain possible explanations rather than conclusions supported by the data.

In general, several factors had differential effects on DS use in physically active men and women. There was no discernible exercise-related factor that differentiated male users from non-users; in contrast, participation in fitness-, strength-, and cardio-oriented activities was the main predictor in women. These sex-specific trends highlight the need for more focused educational initiatives to encourage safe, evidence-based supplement practices while also addressing variations in exercise habits (18).

### 4.2 Factors associated with supplementation frequency

Our findings suggest that supplementation frequency reflects distinct behavioral and motivational profiles, rather than simply different levels of DS use.

Individuals who indicated weekly supplementation or supplementation based on training intensity exhibited a lower likelihood of participating in frequent training compared to daily users. This association may indicate that supplementation frequency corresponds to perceived training requirements, with those engaging in PA more often being more inclined to incorporate daily supplementation into their exercise regimens. Comparable findings have been previously reported, indicating that the use of DSs increases with greater training intensity and competitive participation, signifying elevated nutritional consciousness and perceived physiological requirements among more engaged individuals (1, 4).

Sex differences were also evident. In contrast to daily users, women had a greater propensity than men to indicate weekly supplementation and the use of DSs as meal substitutes. Unfortunately, the present study did not investigate the reasons for these discrepancies, which may indicate varying supplementation approaches rather than disparities in total DS consumption. Women may be more discerning in adopting DS use based on their nutritional practices or particular health goals, while males may be more likely to adhere to consistent supplementation regimens. Nonetheless, these interpretations are conjectural and need investigation in forthcoming qualitative and longitudinal research.

A significant association was observed between supplementation frequency and participants' stated goals and perceptions regarding DSs. Compared with daily users, weekly users were less likely to report compensating for nutritional deficiencies or promoting muscle growth as motivations for DS use and were more likely to report that DSs had no effect on their energy levels. Similarly, participants who used DSs according to training intensity were less likely to report muscle growth as a primary goal, while those using DSs as meal replacements were less likely to identify post-exercise recovery as a purpose of supplementation. These findings indicate that supplementation frequency was associated with differences in reported motivations and perceptions regarding DSs (19). Because the study was cross-sectional, the direction of these associations cannot be established.

Notably, individuals who indicated taking DSs within advised daily dosages were more likely to think that supplements provide nutrients that are not adequately obtained from food. This suggests an enhanced understanding of the supportive function of DSs in a well-rounded diet, rather than perceiving them as performance enhancers. Current consensus statements highlight that DSs complement, not replace, an adequate diet and should be used only when nutritional needs cannot be fulfilled through food consumption alone (3, 20).

Overall, supplementation frequency was associated with differences in training practices and users' reported attitudes toward DSs. Participants who supplemented daily differed from less frequent users in training frequency and several reported motivations and perceptions regarding DS use. These findings suggest that supplementation frequency may reflect different patterns of supplement use and training behavior. However, because this cross-sectional study identified these relationships, subsequent longitudinal research is needed to determine whether changes in training behavior could affect supplementation frequency or if shifting perceptions of supplement efficacy could drive enduring trends in DS consumption.

### 4.3 Factors associated with self-reported side effects

Results of this study suggest that self-reported side effects are linked to specific demographic characteristics and purchasing habits, rather than supplementation behaviors alone.

The inverse association between age and self-reported side effects suggests that younger participants reported side effects more frequently. The mechanisms underlying this association cannot be determined from the present data. Variables such as dosage, duration of use, number and type of supplements consumed, medication use, dietary intake, comorbidities, and previous supplement experience were not assessed and may contribute to the observed association.

Participants with intermediate income levels were more likely to report side effects than those in the highest income category. Unlike age and purchase source, these data do not show a distinct socioeconomic gradient and should be treated with caution. It is possible that differences in purchasing habits, product quality, or access to professional nutritional advice influenced this association, though these factors were not examined in the current study. Alternatively, the observed link may be a chance finding that should be confirmed or refuted in future studies.

Purchasing dietary supplements through internet retailers was independently associated with self-reported side effects. Participants who obtained DSs online were more than three times more likely to report side effects than those who purchased them from authorized sources. This finding is consistent with broader concerns regarding the quality and safety of DSs marketed online. Previous research has shown that adverse events associated with DS use may be related to contamination, undeclared ingredients, excessive dosing, inappropriate use, and interactions with prescription medications (21–23). Products acquired from unlicensed internet sellers may also be more susceptible to quality-control problems, potentially increasing the risk of adverse health outcomes. However, these mechanisms were not assessed in the present study. The present study did not assess product quality, product authenticity, dosage, specific supplement composition, or whether online purchases were obtained from regulated or unregulated vendors. Therefore, the observed association should be interpreted as an association rather than evidence that internet purchasing itself causes adverse effects.

After controlling for potential confounders, supplementation frequency and nutritional fact label-reading behavior had no independent association with self-reported adverse effects. This implies that adverse occurrences are unlikely to be determined entirely by how frequently supplements are ingested or whether users read product labels. Instead, characteristics not considered in this study—such as supplement composition, dose, concurrent use of numerous products, underlying health concerns, and product legitimacy—may be more relevant for evaluating supplement safety.

Overall, the current data highlight that the safe use of DSs is dependent not only on consumer behavior but also on product quality and purchasing habits. Given the growing popularity of online supplement sellers, healthcare professionals and public health officials should encourage consumers to purchase DSs from regulated sources. Professional advice should also be sought before beginning supplementation, especially if consumers are taking multiple products or performance-oriented supplements. Future longitudinal studies that include objective measures of supplement exposure, product composition, and clinically validated adverse events are required to better understand the determinants of DS safety in physically active individuals (24).

### 4.4 Factors associated with nutritional fact label-reading behavior

The current results indicate that the act of reading nutritional fact labels constitutes a distinct health habit linked to certain user traits and beliefs, rather than being related to supplementation practices alone.

Females were almost three times more likely than males to report reviewing nutritional fact labels before using DSs. This finding is consistent with previous research reporting sex-related differences in nutrition- and health-information seeking, although the mechanisms underlying this association were not assessed in the present study (25). Previous studies have reported that women tend to pay greater attention to nutritional fact labels, possibly reflecting greater health awareness and information-seeking behaviors. However, health literacy and nutritional knowledge were not assessed in the present study and therefore cannot be evaluated as explanations for the observed sex difference. These factors may warrant investigation in future research.

The practice of reading nutritional fact labels was also independently linked to employment in the public sector. Due to the lack of assessment of the mechanisms driving this link and the limited availability of comparable evidence, this discovery warrants careful interpretation. Subsequent research ought to examine whether variations in occupational health promotion, accessibility of health information, or other workplace attributes influence this correlation.

A significant discovery was the graded relationship between perceived metabolic improvement and the examination of nutritional fact labels. In contrast to those who indicated no perceived metabolic advantage, those who reported progressive improvements in perceived metabolic effects were more likely to examine product labels. Nonetheless, the cross-sectional design inhibits the ability to ascertain the directionality of this association. It is therefore unclear whether perceived metabolic improvement is related to greater attention to product information, whether label-reading is associated with perceived benefits, or whether both are influenced by other unmeasured factors.

A significant finding was that the act of reading nutritional fact labels did not correlate independently with a reduction in self-reported side effects after controlling for any confounding variables. This discovery aligns with the results of a systematic review conducted by (26); the authors determined that while numerous customers claim to read DS labels, their understanding of the material is frequently inadequate, and enhancements in label design alone have minimal influence on consumer behavior. Interpreting nutritional fact labels does not inherently indicate sufficient nutrition literacy or the capability to accurately understand ingredient lists, dose guidelines, contraindications, or possible interactions with pharmaceuticals and other DSs. Similarly, Lenssen et al. (27) discovered that offering more comprehensive product details heightened consumers' preference to use DSs; this indicates that interaction with label information could affect perceptions and buying choices without necessarily encouraging the responsible or safer use of DSs.

After controlling for potential confounding factors, educational attainment, frequency of supplementation, source of purchase, and self-reported adverse effects were not independently linked to the reading of nutritional fact labels. These results indicate that personal attitudes and perceived advantages have a greater impact on label reading than supplementation habits or other assessed demographic factors. Significantly, the findings suggest that referencing nutritional fact labels ought to be regarded as a factor in responsible DS use, rather than a dependable measure of safe supplementation.

These results indicate that merely reading nutritional fact labels may not be adequate for the safe use of DSs. Public health initiatives should therefore go beyond encouraging people to read product labels, and focus on enhancing nutritional literacy and the capacity to critically analyze ingredient details, suggested dosages, contraindications, and possible combinations. Such educational initiatives could encourage the safe, informed, and evidence-supported use of DSs among physically active individuals.

### 4.5 Strengths and limitations

This study has several strengths. To our knowledge, this is one of the first studies to look at multiple aspects of DS use in a single analytical framework among physically active adults in Saudi Arabia, including factors associated with supplement use, supplementation frequency, self-reported adverse effects, and nutritional facts label-reading behavior. The large sample size (*N* = 755, including 505 supplement users) allowed for multivariable analyzes of many supplementation-related outcomes. Furthermore, the study used a previously constructed and validated questionnaire, and the current analysis was carried out on the same dataset as the published Part I study, allowing the current work to expand on the previously reported descriptive and bivariate results.

However, these findings must be considered in light of several limitations. First, the cross-sectional design prevents temporal or causal inferences about the observed associations. Second, the use of non-probability quota sampling limits generalizability beyond the study population and the Eastern Province of Saudi Arabia. Third, the study relied on self-reported information and was therefore subject to recall and social desirability biases. Fourth, the questionnaire did not collect extensive information on individual supplement dosages, specific products or brands, duration of supplement use, or concurrent use of multiple supplements. These omissions limit the ability to examine dose-related or product-specific associations with self-reported side effects. Fifth, no data regarding concurrent drug usage, dietary intake, pertinent comorbidities, or professional advice or supervision about supplement use were recorded. These unmeasured factors may represent residual confounding, particularly in analyses of self-reported adverse effects and supplementing behaviors. Sixth, self-reported side effects and perceived metabolic enhancement were subjective reports and were not clinically or biochemically confirmed. In particular, perceived metabolic enhancement should not be considered as an objective metric of metabolic change. Seventh, although sparse categories were collapsed prior regression modeling to improve cell frequencies and model stability, recategorization necessarily reduced some of the detail contained in the original questionnaire responses. Finally, the study was conducted in the Eastern Province of Saudi Arabia, and the findings may not generalize to other regions or to non-physically active populations.

### 4.6 Practical implications

The current findings offer significant practical implications for public health policy and the domain of sports nutrition. Considering the widespread consumption of DSs among physically active individuals, public health initiatives should shift from an approach of general discouragement to one focused on harm reduction. Suitable strategies would emphasize evidence-based supplementation, particularly by promoting informed choices, directing suitable product selection, and highlighting safety measures. This is especially vital in Saudi Arabia, where participation in PA for leisure is increasing due to national health promotion efforts (28).

Our findings reveal unique, sex-specific factors that influence DS consumption, suggesting the need for customized educational initiatives tailored to specific demographic groups. The robust relationship between DS consumption and participation in fitness, strength, and cardiovascular activities among women suggests that fitness centers and sports clubs serve as optimal locations for focused, research-backed nutrition education. In contrast, for men, supplementation habits were associated with both PA involvement and work-related elements, requiring a more comprehensive instructional approach that encompasses training methodologies and the intricacies of DS safety.

Moreover, the identified correlation between online shopping and self-reported negative consequences highlights a significant consumer safety issue. Healthcare practitioners, sports nutritionists, pharmacists, and fitness experts should proactively advise individuals to obtain DSs from authorized, trustworthy suppliers, and to pursue professional guidance prior to commencing any regimen, particularly when utilizing various products or performance-enhancing substances. Public health initiatives should emphasize educating the public about the intrinsic dangers linked to items purchased from unregulated online vendors.

Finally, while label-reading behavior was prevalent among our cohort, its lack of an independent association with a reduction in self-reported side effects suggests that this behavior alone is insufficient to prevent harm. Future educational programs should go beyond encouraging label recognition and concentrate on expanding practical nutrition literacy. DS users must possess the ability to analyze ingredient lists, understand recommended doses, and identify contraindications and interactions with pharmaceuticals or other supplements (29). Enhancing this degree of literacy would enable individuals to use DSs more safely and effectively.

## 5 Conclusions

This study presents some of the first evidence on DS behaviors among physically active individuals in the Eastern Province of Saudi Arabia by examining factors influencing DS usage, frequency of supplementation, self-reported adverse effects, and the behavior of reading nutritional fact labels. The use of DSs was prevalent in the population, with over two-thirds of individuals indicating they had used supplements in the past year, highlighting the extensive incorporation of DSs into active lifestyles.

Although the prevalence of supplement use was similar between men and women, the factors associated with dietary supplement use differed according to sex, indicating that supplementation is influenced by distinct behavioral and sociodemographic factors. For women, engagement in fitness, strength, and cardio activities was the only independent factor distinguishing supplement users from female non-users, with nearly fourfold higher odds compared with stationary cycling. Among men, higher training frequency and employment status were associated with sex-specific supplement-use status. Daily supplementation was associated with male sex, higher training frequency, and goal-directed beliefs.

Internet purchase of supplements was associated with, rather than shown to cause, self-reported side effects. Women and public-sector employees were more likely to report reading nutritional labels, and progressively greater perceived metabolic enhancement was associated with higher odds of label-reading behavior. Because the study was cross-sectional, these findings should be interpreted as associations rather than causal effects.

These findings support the need for targeted educational and public-health strategies addressing safe and informed dietary supplement practices among physically active adults. Future longitudinal research should examine supplement dose, specific products, duration of use, concurrent supplement and medication use, dietary intake, professional advice, and objectively assessed adverse events.

## Data Availability

The raw data supporting the conclusions of this article will be made available by the authors, without undue reservation.
