# Supplementary material for: Factors associated with dietary supplement use among physically active adults in Saudi Arabia: an analytical extension of a population-based study
Source: Front Nutr. 2026 Sep 29;13:1955972. doi: 10.3389/fnut.2026.1955972 (PMC13658447; doi:10.3389/fnut.2026.1955972)
Supplement: Supplementary file 1 [file Table_1.pdf]

Supplementary Table S1. Target and achieved sample sizes by sex and age group strata (targets based on gym membership distribution).

Sex	Age Group)	Gym Registry Distribution (%)*	Target (n)†	Achieved (n)	Achieved (% of target)
Male	18–29	24%	**79**	182	230.4%
Male	30–44	23%	**75**	176	234.7%
Male	≥45	4%	**13**	24	184.6%
Female	18–29	31%	**102**	235	230.4%
Female	30–44	15%	**50**	116	232.0%
Female	≥45	4%	**13**	22	169.2%
**Total**	**All**	**100%‡**	**330**	**755**	**228.8%**

Note: *Percentages derived from aggregated membership records of partnering fitness clubs and gyms across the Eastern Province (pre-recruitment data). †Targets were calculated by applying the gym registry proportions to the total planned sample (N=330). All targets are rounded to the nearest integer and sum to exactly 330. ‡Due to rounding of individual percentages, the total sums to 101%; the proportions used for target calculation were standardized to sum to 100%.
