# Supplementary material for: Factors associated with dietary supplement use among physically active adults in Saudi Arabia: an analytical extension of a population-based study
Source: Front Nutr. 2026 Sep 29;13:1955972. doi: 10.3389/fnut.2026.1955972 (PMC13658447; doi:10.3389/fnut.2026.1955972)
Supplement: Supplementary file 2 [file Table_2.pdf]

Supplementary Table S2. Target and achieved sample sizes by recruitment city (based on population proportions).

City	Population Proportion (%)*	Target (n)†	Achieved (n)	Achieved (% of target)
Dammam	39.8%	**131**	224	171.0%
Khobar	17.1%	**56**	146	260.7%
Qatif	14.4%	**48**	103	214.6%
Al-Ahsa	28.7%	**95**	282	296.8%
**Total**	**100.0%**	**330**	**755**	**228.8%**

Note: *Population proportions are based on the combined populations of the four municipalities only (General Authority for Statistics, GASTAT).
†Geographic targets were calculated by applying the respective population proportion to the total planned sample (N=330). All targets are rounded to the nearest integer and sum to exactly 330.
